# Editorial: The impact of chronic stress on neuroplasticity and abnormal behavior

**DOI:** 10.3389/fnbeh.2023.1208351

**Published:** 2023-05-16

**Authors:** Jacob C. Nordman, Cliff Summers, Kevin Ball

**Affiliations:** ^1^Department of Physiology, University of Southern Illinois School of Medicine, Carbondale, IL, United States; ^2^Department of Biology, University of South Dakota, Carbondale, IL, United States; ^3^Department of Psychology, Bloomsburg University, Bloomsburg, PA, United States

**Keywords:** chronic stress, neuroplasticity, abnormal behavior, mental health, innovative treatments

## Introduction

Chronic stress has far-reaching consequences on brain circuits, behavior, and mental health, often leading to abnormal behavior and psychiatric disease. The repercussions of chronic stress affect society as a whole, placing undue strain on the economy and increasing the risk of catastrophic events in communities with easy access to firearms. Developing innovative treatments and preventative measures for stress-induced abnormal behavior requires a more comprehensive understanding of how chronic stress impacts the brain.

Neuroplasticity, the process of brain modification at the cellular and circuit levels, has traditionally been associated with learning and memory. However, recent research reveals its role in altering innate and adaptive behavioral responses, especially those influenced by stress. By inducing plasticity within specific brain circuits, many chronic stress-related effects can be mitigated.

This Research Topic delves into the complex relationship between chronic stress, neuroplasticity, and abnormal behavior. It features original research and review articles that employ novel tools, pharmacological approaches, and various animal models to investigate the potential of augmenting plasticity within brain circuits as a means of treating symptoms caused by chronic stress. Topics span early life stress, synaptic remodeling, and epigenetic factors that increase susceptibility to stress-induced abnormal behavior.

## Overview of the articles in this Research Topic

This Research Topic adopts an interdisciplinary approach, combining research from various disciplines to provide a comprehensive and multifaceted understanding of the relationship between chronic stress, neuroplasticity, and abnormal behavior.

Shi et al. investigates the effects of direct and indirect stress on synaptic plasticity in the anterior insular cortex (aIC) using an observational fear mouse model. The results provide insights into the changes in synaptic plasticity in the aIC after physiological and psychological stress and suggest that different types of stress may have different underlying mechanisms, which could aid in the treatment of stress-related disorders. This study highlights the importance of understanding how various forms of stress impact neural circuits and contribute to abnormal behavior, ultimately informing targeted interventions.

Bartsch and Nordman focuses on understanding the early signs and deterioration of perceived fatigue in healthy middle-aged individuals to develop preventive strategies for chronic fatigue (CF) and chronic fatigue syndrome (CFS). By understanding fatigue development in middle-aged adults, the study aims to contribute to the establishment of countermeasures for CF and CFS. This research underscores the significance of early detection and prevention of fatigue-related disorders, which can have debilitating effects on individuals' quality of life and productivity.

Putra et al. discussed how NMDA receptor antagonists, such as ketamine and memantine, have emerged as promising alternatives for treating aggressive behavior due to their quicker onset of action, fewer observed side effects, and potential to serve as long-lasting treatment options. However, their effects on aggression are highly dependent on dosage, context, and personal experience. A study in this Research Topic explores the complexities of these factors and their impact on the effectiveness of NMDA receptor antagonists in managing aggressive behavior. The findings emphasize the need for further research and careful consideration of individual factors to ensure the responsible use of these drugs and to maximize their benefits while minimizing potential risks.

Lastly, Ronan et al. investigated the impact of intracerebroventricular administration of corticotropin-releasing factor (CRF) and vasopressin (AVP) on extracellular serotonin release in the central nucleus of the amygdala (CeA), shedding light on potential mechanisms underlying stress-induced affective reactivity in humans. This knowledge may prove useful in understanding and treating stress-related psychiatric disorders, such as anxiety, depression, and posttraumatic stress disorder. By examining the neurochemical changes associated with stress-induced affective reactivity, this research advances our understanding of the complex relationship between chronic stress and mental health disorders.

This broad scope offers a more complete picture of the complex relationship between chronic stress and mental health, with promising implications for the development of novel therapeutic interventions and preventative measures for stress-related disorders.

## Innovative methodologies

The Research Topic features innovative methodologies to study the relationship between chronic stress, neuroplasticity, and abnormal behavior. *In vivo* pharmacological manipulations enable targeted drug administration to the brain, while novel behavioral paradigms, such as the observational fear mouse model, allow for exploration of different stress types on brain circuitry. Assessments of individuals in highly social and stressful environments emphasize the importance of early detection and prevention. Direct manipulations of plasticity in live animal models offer targeted approaches to understanding stress-induced effects. Examining the consequences of pharmacological interventions for treating stress-related aggression provides insights into potential benefits and risks. These cutting-edge methods significantly contribute to ongoing efforts in developing innovative treatments and preventative measures for stress-induced abnormal behavior.

## Conclusion and call to action

This Research Topic's implications are significant, contributing to our understanding of chronic stress and its impact on mental health while pointing to novel therapeutic interventions for stress-related disorders. The featured studies explore various aspects of the complex relationship between chronic stress, neuroplasticity, and abnormal behavior. They examine the effects of stress on brain circuitry, neurochemical changes, and early signs of fatigue, as well as the potential of NMDA receptor antagonists to treat aggressive behavior. Collectively, these studies contribute to a holistic understanding of the impact of chronic stress on mental health and wellbeing.

As our understanding of the relationship between chronic stress, neuroplasticity, and abnormal behavior deepens, we become better equipped to address the pressing challenges of mental health and support individuals and society as a whole in coping with the often devastating consequences of chronic stress.

We hope this collection of articles serves as a catalyst for further research and collaboration among scientists, clinicians, and policymakers, driving the development of innovative and personalized interventions for those suffering from stress-related disorders. Together, we can work toward a future where the adverse effects of chronic stress are better understood, managed, and ultimately, mitigated. With continued research and collaboration, we can make strides in developing effective treatments and preventative measures for stress-induced abnormal behavior, improving mental health outcomes and the overall quality of life for individuals affected by chronic stress.

## Author contributions

All authors listed have made a substantial, direct, and intellectual contribution to the work and approved it for publication.

